# The Effects of Flavonoids on Cardiovascular Health: A Review of Human Intervention Trials and Implications for Cerebrovascular Function

**DOI:** 10.3390/nu10121852

**Published:** 2018-12-01

**Authors:** Amy Rees, Georgina F. Dodd, Jeremy P. E. Spencer

**Affiliations:** Department of Food and Nutritional Sciences, University of Reading, Whiteknights, Reading RG6 6AP, UK; a.j.rees@pgr.reading.ac.uk (A.R.); g.dodd@reading.ac.uk (G.F.D.)

**Keywords:** flavonoid, cocoa, blood pressure, flow-mediated dilation, cerebral blood flow, cardiovascular disease, cerebrovascular function

## Abstract

Research has suggested a number of beneficial effects arising from the consumption of dietary flavonoids, found in foods such as cocoa, apples, tea, citrus fruits and berries on cardiovascular risk factors such as high blood pressure and endothelial dysfunction. These effects are thought to have a significant impact upon both vascular and cerebrovascular health, ultimately with the potential to prevent cardiovascular and potentially neurodegenerative disease with a vascular component, for example vascular dementia. This review explores the current evidence for the effects of flavonoid supplementation on human endothelial function and both peripheral and cerebral blood flow (CBF). Evidence presented includes their potential to reduce blood pressure in hypertensive individuals, as well as increasing peripheral blood perfusion and promoting CBF in both healthy and at-risk populations. However, there is great variation in the literature due to the heterogeneous nature of the randomised controlled trials conducted. As such, there is a clear need for further research and understanding within this area in order to maximise potential health benefits.

## 1. Cardiovascular Health and Flavonoids

Cardiovascular disease (CVD) is one of the main causes of death worldwide, yet it is largely preventable [1]. Although there are now treatments available, focus should be on prevention of disease through reduction of risk factors which could be achieved by encouraging healthy lifestyle choices. A further implication of CVD is its link with neurodegenerative diseases of a vascular aetiology such as vascular dementia [2,3]. Dementia is a growing concern worldwide as people are living longer, and a condition for which we currently do not have any effective treatments. Therefore, prevention of CVD and its risk factors is not only important for vascular health but also cerebrovascular health. The impact of diet on such diseases is of particular interest with emerging research suggesting that dietary flavonoids may have cardio- and neuro-protective effects [4,5,6], mediated by their interactions with the vascular system [7,8,9,10]. However, to date, the precise mechanisms by which their effects are mediated in vivo and what doses are required to induce such effects remain unclear.

Flavonoids are naturally occurring compounds found in a variety of fruit, vegetables, and plant-based food products and represent the second largest group of polyphenols present in the human diet. Flavonoids can be divided into several subclasses, based upon variations in the structure, with the basic structure allowing for a large number of different substitutions in the A, B, and C rings. Subclasses include flavonols, flavanones, anthocyanins, flavones, isoflavones, and the most common subclass, flavanols. Flavanols exist in both the monomer form as catechins and the polymer form as proanthocyanidins, and are found predominantly in cocoa, apples, and tea [11]. Only a small proportion of flavonoids ingested are absorbed intact, and the rate and extent to which they are absorbed varies greatly on a number of factors such as the structure of the molecule, the matrix in which it is bound and interactions with other components, as well as inter-individual differences such as age, sex, and composition of the colonic microbiota [12]. Once ingested, flavonoids pass through the stomach and into the small intestine where phase I metabolism occurs. Following absorption in the epithelial cells of the small intestine, flavonoids undergo phase II metabolism to form conjugated metabolites which enter the circulation via the portal vein [13]. However, only an estimated 5–10% of total polyphenol intake is absorbed in the small intestine [14], with the remaining larger flavonoids continuing on to the large intestine where the colonic microflora are able to degrade them into low-molecular-weight metabolites which can be absorbed. Once in the bloodstream, metabolites are transported around the body whereby they can act on the relevant tissues or are transported to the liver for further metabolism before ultimately being excreted [13]. The large variation in bioavailability of flavonoids can make it more difficult to study the effects they have within the body and can lead to discrepancies in the literature.

**Figure 1:**
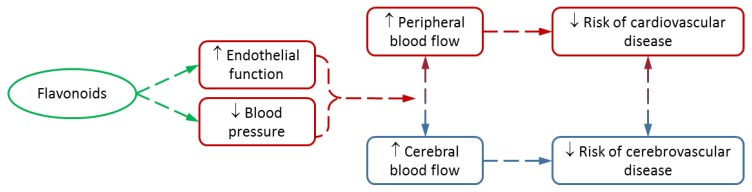
The effects of flavonoids on vascular and cerebrovascular function and implications for health.

Vascular function has been shown to be linked with cognition and brain function, with increased cardiovascular health being associated with greater cognitive performance [15,16,17]. Furthermore, many of the risk factors associated with cardiovascular health are also risk factors for cerebrovascular health, such as hypertension, hypercholesterolemia, and diabetes, with CVD itself having been identified as a risk factor for vascular dementia, caused by a reduction in blood flow to the brain [18,19]. Therefore, it is important to understand the effect flavonoids have upon the vascular system in order to fully understand the effect that they are also having on the brain (Figure 1).

## 2. Epidemiological Evidence

Recent epidemiological studies have suggested a positive association between diets high in flavonoid-rich foods and cardiovascular health [20,21,22,23,24,25]. An 18% reduction in the risk of fatal CVD in those with total flavonoid intakes in the top quintile (≥359.7 mg/day) compared with those in the bottom quintile (<121.5 mg/day) has been demonstrated and it has been suggested, due to the non-linear nature of many of the associations observed, that even a relatively low habitual intake of flavonoid-rich foods may be beneficial in reducing the risk of fatal CVD [20]. An association between reduced risk of death due to CVD and dietary intake of flavanones, anthocyanidins and certain flavonoid-rich foods such as apples, red wine, grapefruit and chocolate was also found [21]. There have been a number of prospective studies which have highlighted in particular the association between higher chocolate, cocoa, or epicatechin intake and a lower risk of CVD mortality and future cardiovascular events [26,27,28]. Despite the majority of these studies suggesting a greater habitual intake of flavonoid-rich foods is associated with a lower risk of CVD, they cannot prove cause and effect due to the uncontrollable nature of observational studies. Methods of dietary assessment employed are not reliable, especially with huge variability in the flavonoid content of foods due to differences in growing and processing conditions, along with many other variables which cannot be controlled. These studies provide a good starting point for further research, but carrying out clinical trials is the only way to fully understand the effects flavonoids can have in the human body. Therefore, this review will explore the current clinical evidence from human intervention randomised controlled trials for the effects of flavonoid-rich foods, with a particular emphasis on cocoa, on vascular and cerebrovascular health.

## 3. Impact of Flavonoid Consumption on Blood Pressure

Blood pressure is an important predictor of cardiovascular health, with a lower blood pressure being linked to better vascular health [29,30]. On a population level, a reduction in systolic blood pressure of only 2 mmHg may result in a 10% lower stroke mortality and 7% lower mortality from ischaemic heart disease and other vascular causes [31]. As such, the impact of flavonoid-rich food consumption, cocoa in particular, on blood pressure has been studied fairly extensively. A reduction of 4.4 mmHg and 3.9 mmHg in systolic and diastolic blood pressure respectively, was demonstrated following consumption of a cocoa flavanol drink containing 900 mg total flavanols per day for 1 month compared with a control [32]. Similarly, another trial observed a reduction in blood pressure (systolic: −4.8 mmHg; diastolic: −3 mmHg) following consumption of 10 g cocoa containing various doses of flavonoids up to 800 mg (0, 80, 200, 500 and 800 mg flavonoids) over the period of a week in healthy adults [33]. A study investigating the effects of a cocoa flavanol drink containing 450 mg total flavanols in healthy younger and older males found a significant decrease in systolic blood pressure in the older group following acute consumption and daily intake over 2 weeks compared to baseline, but not in the younger group, although the latter had reductions in diastolic blood pressure over an acute timeframe [34]. At baseline, blood pressure was significantly greater in the older group suggesting that either the younger group were too healthy for any effects to be seen or that flavonoids have a greater effect in at-risk populations, whose vascular health is not at its optimum, and therefore, may benefit most. Similar studies in at-risk populations, such as those with cardiovascular risk factors, have also found positive effects. Flavanol-rich dark chocolate (821 mg flavanols) led to a reduction in blood pressure (systolic: −3.2 mmHg; diastolic: −1.4 mmHg) 2 h post consumption in overweight adults [35]. The same study also found a reduction in blood pressure with a sugar free cocoa drink (805 mg flavanols), however, there was no difference between the sugared cocoa drink (605 mg flavanols) and the control, demonstrating that the beneficial effect of flavonoid-rich cocoa can be negated by the detrimental effect of sugar in the diet. Observations such as these can help us to understand the best form in which to administer cocoa flavanols in order to maximise the health benefits. Significant reductions of 5.3 mmHg and 3 mmHg in systolic and diastolic blood pressure respectively, were observed in subjects with mild, untreated hypertension following cocoa consumption over the course of six weeks, but only in the highest dose administered of 1052 mg cocoa flavanols per day [36]. In contrast to this, a similar study showed significant reductions in systolic (−2.9 mmHg) and diastolic (−1.9 mmHg) blood pressure following a much lower daily intake of only 30 mg cocoa flavanols per days for 18 weeks in mildly hypertensive patients, perhaps due to the longer supplementation period [37]. The authors also stated that the magnitude of reduction in blood pressure was greater in those with higher blood pressure at baseline, again supporting the idea that the benefit of flavonoid supplementation is greater in those with impaired vascular function. Significant reductions in blood pressure were also found in elderly individuals with [38] and without [39] mild cognitive impairment when consuming 520 mg or 993 mg of cocoa flavanols per day compared with a low dose of 48 mg. However, there are a number of studies which have found no effect of cocoa flavanol intake on blood pressure in healthy volunteers, possibly due to the dosage being too low to elicit effects [40,41,42], the time frame for supplementation being too short [43], the use of pure epicatechin rather than a whole food [44] or because effects are more likely to be seen in at-risk populations as mentioned previously.

The effects of a number of other flavonoid-rich foods on blood pressure have also been investigated, although to a lesser extent than cocoa. Consumption of flavanol-rich apple, containing 180 mg (-)-epicatechin, resulted in a reduction in systolic blood pressure of 3.3 mmHg in healthy adults [45]. On the other hand, no significant effect was found following consumption of apple containing 48 mg epicatechin compared to a low-flavanol apple [46]. This may be due to the flavanol content being quite low, comparable to the levels used in control products in other studies [38,39], although the total polyphenol content was greater at 306 mg/day. Black tea, also rich in flavanols, particularly catechins, has been found to reduce both systolic (−2.6 mmHg) and diastolic (−2.2 mmHg) blood pressure following daily consumption for 1 week in healthy males [47]. Chronic consumption of three cups per day (429 mg total polyphenols) for six months also reduced blood pressure to a similar extent in subjects with normal or slightly raised blood pressure [48]. Further reductions in blood pressure (systolic: −3.2 mmHg; diastolic: −2.6 mmHg) were observed in hypertensive subjects following consumption of black tea containing 258 mg flavonoids per day for a week, as well as the ability of acute consumption to prevent an increase in blood pressure following a high fat load [49]. High fat meals have been found to increase postprandial blood pressure [50], and as much of the day is spent in the postprandial state, relieving some of this stress on the vascular system could help to improve overall vascular health. However, a similar study found an increase in blood pressure following black tea consumption which was counteracted by consuming a meal alongside the tea [51]. Further research is required in order to understand these conflicting studies, but in general, black tea appears to have a positive effect by reducing blood pressure in both healthy and hypertensive subjects.

The effects of anthocyanin-rich berries and flavanone-rich citrus fruitson blood pressure have not been so convincing or as promising. A significant reduction in systolic blood pressure (−6 mmHg) was observed in individuals with metabolic syndrome consuming polyphenol-rich grape powder for 30 days compared with a control [52]. However, no significant effects were found following consumption of blueberry juice [53], cranberry juice in healthy subjects [54] or subjects with coronary artery disease (CAD) [55], and elderberry anthocyanins in postmenopausal women [56]. With respect to flavanone-rich foods, orange juice was shown to significantly reduce diastolic blood pressure in overweight males [57], yet no effects were found following consumption of orange juice in healthy males [58], or grapefruit juice in healthy, postmenopausal women over a six month period [59]. Perhaps the effects of flavanones are lesser, and therefore, only visible in those at risk of CVD, explaining the reduction seen in blood pressure in overweight subjects but not in healthy subjects.

It is evident from these studies that flavanol-rich foods such as cocoa and tea, are able to lower blood pressure in healthy as well as at-risk populations, such as those with hypertension and impaired cardiovascular function. It is likely that improvements in endothelial function are responsible for reductions observed, as discussed in greater detail elsewhere [60]; however, flavonoids may also be able to directly affect blood pressure, for example, by inhibiting angiotensin-converting-enzyme (ACE) activity [61]. Studies which observed no change in blood pressure perhaps used too low a dosage of flavonoids, or too short a supplementation period, indicating that longer term supplementation may be required in order to elicit significant effects. Overall, current evidence demonstrates a positive effect of flavanols on blood pressure, but further research is required in order to elucidate the optimal dose and time frame in which flavanols might reliably reduce blood pressure. On the other hand, the evidence is not so clear cut with respect to anthocyanins and flavanones, perhaps due to a lack of studies. It is also possible that these subclasses of flavonoids are not able to modify blood pressure, or the effects are smaller, and therefore, only apparent in those with compromised vascular function. However, this theory would need to be substantiated with further research and evidence. See Table 1 for a summary of studies assessing the impact of flavonoids on blood pressure.

## 4. Impact of Flavonoid Consumption on Endothelial Function

There is an increasing body of evidence from randomised, controlled clinical trials suggesting that flavonoids may be beneficial for the vascular system, particularly with regard to the prevention of endothelial dysfunction [41,45,47,53]. Endothelial function can be described as arterial vasomotor responses mediated by the release of vasodilatory and vasoconstricting chemicals from the endothelium [62]. An imbalance in these endothelium-derived relaxing and contracting factors results in endothelial dysfunction, most commonly characterised by the impaired release of the vasodilator, nitric oxide (NO), predisposing the vasculature to vasoconstriction [63,64,65]. A reduction in NO bioavailability may occur as a result of reduced production by endothelial nitric oxide synthase (eNOS) or increased breakdown by reactive oxygen species [66]. Endothelial dysfunction, considered to take place early on in the pathology of vascular disease, contributes to atherosclerotic plaque formation and has been found to correlate with future cardiovascular events [64,67]. Endothelial function (or dysfunction) can be measured non-invasively using a technique called flow-mediated dilation (FMD), the current preferred method for assessing endothelial function [68]. Increases in FMD, and therefore improvements in vascular function, have been demonstrated following the consumption of a wide variety of flavonoid-rich foods and derived beverages, including cocoa, blueberries, black tea, and apples [32,35,41,45,46,52,53,58,69]. Through measuring endothelial function, FMD effectively reflects NO bioavailability in vivo, thus an improvement in FMD response following flavonoid consumption would suggest an increase in the levels and activity of eNOS, a major source of NO in the endothelium [3,70,71].

Flavanol-rich cocoa in particular has been studied for its potentially beneficial effect on endothelial function with FMD response having been shown to improve, and therefore reduce cardiovascular risk, in a number of human intervention studies following acute and chronic supplementation [32,35,43,72]. In a study looking at acute consumption of a single dose of dark chocolate (821 mg total flavanols) in 45 overweight subjects, an increase in FMD response of 4.3% was observed compared with the control after 2 h [35]. Acute dark chocolate consumption, containing a lower dose of 395 mg total flavanols, was found to increase FMD response by 2.4% compared with milk and white chocolate [73]. A significant increase in FMD response was also found in 10 healthy volunteers following consumption of a single cocoa beverage, containing 917 mg flavanols, up to 4 h post consumption [72]. A simultaneous increase in nitroso species concentrations was observed up to 3 h post consumption, supporting the theory that cocoa flavanols are able to improve FMD response through the activation of eNOS. Further to this, subjects were given solutions containing 1 mg/kg and 2 mg/kg body weight pure epicatechin and FMD was found to significantly increase with both doses, compared with baseline and the control, to a similar level as that found with high flavanol cocoa [72]. This suggests that the beneficial effects of cocoa on endothelial function are related, at least in part, to epicatechin content. A clear limitation of this study was that it was carried out in only three subjects but as a proof-of-concept study it appears promising. However, a similar study looking at pure epicatechin supplementation over a four week period found no significant change in FMD and suggested that epicatechin was not responsible for the observed vascular effects of cocoa [44]. Following on from this, acute consumption of dark chocolate was found to significantly increase FMD compared to the control whereas pure epicatechin did not produce a significant effect [42]. The lack of effects observed with pure epicatechin may be due to the lower doses used in both studies as volunteers consumed only 100 mg (-)-epicatechin per day. Epicatechin has been found to increase NO at doses of 200 mg [74] and so the dosage of 100 mg may not have been high enough to elicit effects on endothelial function. Furthermore, in the latter study pure epicatechin was consumed with white chocolate which contains more sugar and less theobromine and magnesium than dark chocolate, all of which may have contributed to the lack of effect [42].

Other studies have considered the effects that may arise following longer-term supplementation with cocoa flavanols. Flow-mediated dilation response increased following daily supplementation with a high flavonoid (259 mg total flavonoids) chocolate compared with a control over two weeks, with a mean change of 1.3% in the high flavonoid group [41]. Similar increases in FMD response of 1.2% were found following daily consumption of a cocoa drink containing 900 mg total flavanols over the period of a month [32]. Whilst these do not appear to be large improvements in FMD, it is a promising response, as a 1% increase in FMD has been associated with a 13% lower risk of cardiovascular event at population level [75]. Flavanol-rich cocoa (821 mg) consumption was also found to significantly increase FMD response over a five-day period in younger (<50 years) and older (≥50 years) population groups; however, the effect observed was greater in the older population [43]. This corresponds with studies on blood pressure [34], suggesting that there may be a greater benefit of flavanol supplementation in advancing age, when endothelial function is more likely to be impaired and vascular health is not optimum. A study looking at the effects of apples, also rich in flavanols, on endothelial function in healthy subjects demonstrated a greater FMD response and increased levels of circulating nitroso species following flavonoid-rich apple (180 mg (-)-epicatechin and 184 mg quercetin) consumption [45]. More recently, the beneficial effect of apples on endothelial function was demonstrated in individuals at risk of CVD with a significant increase in FMD following both acute (0.8%) and four weeks of chronic (0.5%) consumption of high flavonoid apples (48 mg epicatechin) [46]. Although this significant increase in FMD is not substantial, as discussed previously, even moderate increases in FMD may have a significant effect on vascular health and the prevention of CVD. On the other hand, daily consumption of apple polyphenol extract, containing 100 mg epicatechin, over a four week period had no significant effect on FMD response in borderline hypertensives compared with the control [76]. Flow-mediated dilation response was measured one and a half hours post consumption of the intervention; as other studies have found improvements in endothelial function at 2 h, FMD may have been measured too early to observe a significant effect. In summary, current research does not lead to a clear conclusion on the acute effects of FMD and flavanol supplementation due to conflicting evidence. Acute studies, supplemented with higher levels of cocoa flavanols, observed positive effects [35,72], whilst those supplementing with lower levels did not [44]. The chronic effects are more promising with little conflicting evidence, even when considering the dosage administered. It may therefore follow that a larger dosage is required to elicit an immediate effect in acute studies, perhaps due to the differing rates of absorption of flavonoids following ingestion, or due to immediate effects not being as powerful as those which have arisen due to chronic supplementation; however, further research would be required to substantiate these theories. Overall, the evidence from existing studies would imply flavanols can improve blood flow in the periphery, through increasing levels of NO available in the endothelium, providing an efficacious dose is administered.

Research into the effects of other flavonoid-rich foods on endothelial function has also revealed promising effects. Black tea was found to improve FMD response in a dose-dependent manner with as little as 100 mg of flavonoids per day, equivalent to less than one cup of tea, being found to increase FMD compared with the control [47]. A significant improvement in FMD response was also observed following both acute and one week daily consumption of black tea compared with hot water [69], whilst acute consumption of both green and black tea was found to significantly improve FMD response by 5% and 4.4% respectively, compared with a control in healthy adults [77]. These studies demonstrate the beneficial effects which could be achieved with the incorporation of a realistic dose of flavonoid-rich black or green tea into the diet. Black tea consumption has also been found to improve fasted FMD response in patients with CAD [78] and in hypertensives [79]. Black tea was also able to counteract the impairment to FMD response with a high-fat challenge in hypertensives [79]. High-fat meals have been found to impair endothelial function postprandially [80] and so the ability of flavonoids to prevent this demonstrates the beneficial effects they are able to exert when the vascular system is compromised or put under stress.

Anthocyanin-rich foods such as grapes, blueberries, and cranberries, and their derivatives, have also been studied but with mixed outcomes. No effects of wine grape or grape seed (800 mg total polyphenols) on FMD were observed over a three-week period in healthy males [81]; however, significant increases in FMD were found following consumption of a grape polyphenol for 30 days in men with metabolic syndrome [52], and consumption of purple grape juice for 14 days in patients with CAD [82]. The conflicting results of these studies suggests that the effects of some flavonoid-rich foods may be more significant in patients and those at risk of developing disease rather than healthy individuals. Blueberries have been shown to significantly increase FMD response in healthy adults with peaks at 1–2 and 6 h post ingestion of polyphenol-rich blueberry drinks [53]. The study demonstrated a linear increase in FMD response, plateauing at a dose of 766 mg total polyphenols. This suggests that there is an optimum dose and that too great an intake may even negate some of the positive effects. A reduction in neutrophil NADPH oxidase activity coincided with increases in FMD, suggesting this as a potential mechanism of action whereby superoxide generation is reduced and NO availability is greater [53]. Furthermore, the beneficial vascular effects of cranberries have been demonstrated, with dose-dependent improvements in FMD response following consumption of cranberry juice, peaking at 4 h and with a maximal effect with juice containing 1238 mg total polyphenols [54]. Further studies into the dose-dependent effects of various flavonoids would be extremely useful when looking to maximise health benefits. However, there were no effects on FMD response observed following consumption of cranberry juice (835 mg total polyphenols) for four weeks in patients with CAD [55]. These two studies used similar doses of cranberry polyphenols; however, the latter measured FMD response in fasted subjects. This suggests that whilst anthocyanins may be able to exert effects whilst they remain in the blood stream, they may be unable to wield any long standing effects. If this is indeed the case, this would be important when considering how best to utilise anthocyanins for the prevention of disease, as perhaps intake should be spread across the day in order to have the optimum effect.

Another subclass of flavonoids, flavanones, found commonly in citrus fruits, have also been of interest. Flavanone-rich orange juice was able to prevent postprandial endothelial function in healthy males with FMD response returning to baseline levels and plasma nitrite levels remaining constant compared with a control drink [58]. This study demonstrates a positive effect by ameliorating postprandial endothelial dysfunction following a high-fat meal, again demonstrating the potential of flavonoids to have greater effects in situations in which vascular health is compromised. The flavanone hesperidin has also been found to improve endothelial function with a daily 500 mg dose for three weeks, improving FMD response by 2.5% in individuals with metabolic syndrome [83]. On the other hand, FMD response remained unchanged in postmenopausal women following a six month supplementation period with grapefruit juice [59]. As discussed previously, this may be due to endothelial function being measured in fasted subjects. Hesperidin was able to elicit effects in fasted subjects [83], but perhaps the improvement in FMD observed was due to the population of the study being at risk, or the pure flavanone being able to elicit a stronger effect. Either way these conflicting studies demonstrate the need for further research in this area.

It is evident that flavonoids have the potential to improve FMD response, and therefore, endothelial dysfunction in both healthy and at-risk populations, as well as when the vascular system is under stress, for example, following a high fat or sugar meal. There is a greater body of evidence with respect to the effects of anthocyanins and flavanones on FMD response, unlike with blood pressure, and current research suggests that all subclasses of flavonoids may be able to enhance endothelial function. However, it is clear that a greater understanding is required of how flavonoids work in vivo and how to incorporate them into the diet in order to fully capitalise on the potential benefits they could have on endothelial function and the vascular system, and their potential to reduce the risk of CVD. See Table 2 for a summary of studies assessing the impact of flavonoids on endothelial function.

## 5. Impact of Flavonoid Consumption on Cerebral Blood Flow

Whilst there is now a fairly large body of evidence for the effect of flavonoids on vascular health and blood flow in the periphery, effects of flavonoids on cerebrovascular health and blood flow in the brain is an emerging area of research. Evidence suggests that flavonoids may have a neuroprotective effect, with the potential to slow the cognitive decline typically associated with ageing [7]. The mechanisms behind these neuroprotective effects are thought to be similar to those acting in the periphery, for example, greater bioavailability of NO, and thus optimal blood flow [84,85]. Increased levels of NO in the cerebrovascular system may improve blood flow throughout the brain, thus inducing neurogenesis in the dentate gyrus of the hippocampus, promoting nerve cell growth and leading to changes in neuronal morphology [86,87]. It is possible that flavonoids, if capable of entering the brain [88,89,90], are also able to improve synaptic plasticity and communication whilst preventing neuroinflammation, ultimately demonstrating an overall neuroprotective effect [13,91]. These combined effects are thought to result in the prevention, or perhaps even the slowing down of progression, of neurodegenerative diseases such as vascular dementia [6].

The flow of blood to and from the brain, known as cerebral blood flow (CBF), provides the brain with a constant supply of glucose and oxygen, and therefore, adequate CBF is essential for the normal functioning of the brain; an insufficient supply of energy will ultimately result in neuronal damage [92,93]. Cerebral hypoperfusion occurs naturally as part of the ageing process but cardiovascular risk factors may promote further reductions in CBF, demonstrated particularly in areas of the brain such as the hippocampus and anterior cingulate cortex thought to precede neurodegenerative disorders such as vascular dementia and Alzheimer’s disease [94,95,96,97]. A number of studies have now demonstrated a beneficial effect of flavonoid-rich foods on CBF and although the precise mechanisms are not yet known, it is thought that this can occur through similar mechanisms to those acting when peripheral blood flow is increased [98,99,100].

There is a growing body of evidence for the effects of flavonoid-rich cocoa on CBF and cocoa has been found to improve cognitive function, perhaps through improving blood flow around the body and increasing the flow of blood to the brain [98,101]. An increase in CBF was observed following consumption of a high flavanol (516 mg) cocoa drink compared with a low flavanol (39 mg) drink, which peaked at 2 h and returned to baseline after approximately 6 h [98]. This study was only carried out in four volunteers; however, a similar study also observed a significant increase in regional perfusion at 2 h, particularly in the anterior cingulate cortex and central opercular cortex of the parietal lobe, when given a flavanol-rich (494 mg) cocoa drink compared with a low flavanol (23 mg) cocoa drink [102]. The increases in CBF with a peak reported at 2 h in these studies corresponds with effects seen on FMD response in studies of vascular function [35]. With regards to longer term supplementation, studies have indicated an increase in blood flow following consumption of flavanol-rich (450 mg and 900 mg) cocoa drinks for 1–2 weeks in healthy older adults [103,104]. Beneficial effects on blood flow were also demonstrated over a three-month supplementation period with 900 mg cocoa flavanols per day; dentate gyrus function was shown to be enhanced in healthy older adults when compared with those following a low flavanol diet [105]. In contrast, dark (394 mg total polyphenols) and milk (200 mg total polyphenols) chocolate consumption was found to significantly lower CBF response during cognitive tasks with no implications for cognitive function when compared with white chocolate [73]. Maintenance of cognitive ability would suggest that there may be other mechanisms involved, allowing the brain to function more efficiently with reduced blood flow. However, not all data supports the beneficial effects of cocoa flavonoids on CBF. A study looking into the acute and sub-chronic effects of a cocoa supplement containing 250 mg of catechin polyphenols found no significant changes in blood flow to the brain, perhaps due to the dosage administered being too low to elicit any benefit or because of the method used, transcranial Doppler sonography, is not as sensitive as others [40].

A number of studies have also been carried out with other flavonoid-rich foods, albeit with mixed results. Blueberries have been found to increase regional perfusion, with an increase in CBF being observed in the precentral and middle frontal gyrus of the frontal lobe and the angular gyrus of the parietal lobe following consumption of a flavonoid-rich (579 mg) blueberry beverage compared with a control [99]. Further to this, a more recent study in healthy adults also found increases in regional perfusion, specifically in the parietal and occipital lobes following 12 weeks of supplementation with a blueberry concentrate containing 387 mg anthocyanins [106]. Increases in regional perfusion in the interior and middle right frontal gyrus have also been observed 2 h post consumption of a citrus drink containing 70.5 mg flavanones [100]. On the other hand, a reduction in CBF to the frontal cortex in healthy adults was observed following a 135 mg dose of pure epigallocatechin gallate (EGCG), the main catechin found in green tea, and no effect with a 270 mg dose when compared with the control [107]. The authors suggest that EGCG may not have a straightforward dose response profile, thus explaining the variation in results seen here arising from different dosages. In addition, it may be that pure compounds do not always have the same effect as can be seen when ingested as a whole food and there may be beneficial synergistic effects at play. It may also be that EGCG improves other aspects of brain function, thus reducing the need for blood flow in the frontal cortex as discussed previously with cocoa [73,107].

Whilst there is some conflicting evidence, it would seem that current research tends to demonstrate improvements in regional perfusion, particularly with regard to acute flavonoid supplementation. It may also be possible for flavonoids to optimise cerebrovascular function in certain regions, in turn reducing CBF. Further research into this effect, the specific regions of the brain which are affected and the effects of chronic consumption would be useful, along with more studies linking the effects of flavonoids in the periphery with those in the brain as there is only one to date [73]. Additional research is essential in order to further our understanding of how flavonoids may be able to exert neuroprotective effects and their potential to prevent the development of neurodegeneration. See Table 3 for a summary of studies assessing the impact of flavonoids on cerebral blood flow.

## 6. Conclusions

Current research suggests that flavonoids are able to exhibit cardio and neuroprotective effects, as demonstrated with improvements in FMD response, reductions in blood pressure and increases in CBF, all effects which can be translated into reductions in the risk of disease. However, the changes observed are not yet fully understood and there are currently discrepancies in the literature. This is largely due to the heterogeneity of intervention studies in terms of the study design, population observed, and duration of the intervention period. Future studies should investigate the dose and form of flavonoid administered in order to elucidate the optimal dose and explore the potential synergistic effects of whole foods as opposed to pure compounds. With regards to cerebrovascular health, research should focus on the areas of the brain in which flavonoids seem to demonstrate the greatest effects in order to have a better understanding of the mechanisms of action and how to target these areas in order to achieve the optimum benefit. Further research in these areas would help us to understand the beneficial effects that the incorporation of daily consumption of flavonoids are able to have on human health, particularly with regard to potentially preventing CVD and neurodegenerative diseases such as vascular dementia.

## Figures and Tables

**Table 1:** Summary of studies investigating the effect of flavonoids on blood pressure.

Author	Flavonoid Source and Dose	Duration	Sample	Effects
Sansone et al. (2015) [32]	Cocoa 900 mg flavanols	1 month	Healthy subjects (*n* = 100)	−4.4 mmHg SBP, −3.9 mmHg DBP
Grassi et al. (2015) [33]	Cocoa 80, 200, 500, 800 mg flavonoids	1 week	Healthy subjects (*n* = 20)	−4.8 mmHg SBP, −3 mmHg DBP
Heiss et al. (2015) [34]	Cocoa 450 mg flavanols twice/daily	Acute and 2 weeks	Healthy younger (aged <35 year, *n* = 22) and older males (aged 50−80 year, *n* = 20)	−5 mmHg SBP (acute) and −6 mmHg SBP (chronic) in older group
Faridi et al. (2008) [35]	Dark chocolate 821 mg flavanols	Acute	Overweight subjects (*n* = 45)	−3.2 mmHg SBP, −1.4 mmHg DBP
	Cocoa 805 mg flavanols (sugar-free), 605 mg flavanols (sugared)	Acute	Overweight subjects (*n* = 45)	−2.1 mmHg SBP, −1.2 mmHg DBP; no effect of sugared cocoa
Davison et al. (2010) [36]	Cocoa 33, 372, 712, 1052 mg flavanols	6 weeks	Mildly hypertensive subjects (*n* = 52)	−5.3 mmHg SBP, −3 mmHg DBP at highest dose, no other effects
Taubert et al. (2007) [37]	Cocoa 30 mg total polyphenols	18 weeks	Mildly hypertensive subjects (*n* = 44)	−2.9 mmHg SBP, −1.9 mmHg DBP
Desideri et al. (2012) [38]	Cocoa 48, 520, 993 mg flavanols	8 weeks	Elderly subjects with MCI (*n* = 90)	−10 mmHg SBP, −4.8 mmHg DBP
Mastroiacovo et al. (2015) [39]	Cocoa 48, 520, 993 mg flavanols	8 weeks	Elderly subjects (*n* = 90)	−7.8 mmHg SBP, −4.8 mmHg DBP
Massee et al. (2015) [40]	Cocoa 250 mg polyphenols	Acute and 4 weeks	Healthy subjects (*n* = 40)	No significant effect
Engler et al. (2004) [41]	Cocoa 213 mg procyanidins, 48 mg epicatechin	2 weeks	Healthy subjects (*n* = 22)	No significant effect
Dower et al. (2016) [42]	Dark chocolate 150 mg epicatechin, 100 mg pure epicatechin with white chocolate	Acute	Healthy males (*n* = 20)	No significant effect
Fisher and Hollenberg (2006) [43]	Cocoa 821 mg flavanols	4−6 days	Healthy younger (aged<50 year, *n* = 15), and older subjects (>50 year, *n* = 19)	No significant effect
Dower et al. (2015) [44]	Pure epicatechin 100 mg	4 weeks	Healthy subjects (*n* = 37)	No significant effect
Bondonno et al. (2012) [45]	Apple 180 mg epicatechin, 184 mg quercetin	Acute	Healthy subjects (*n* = 30)	−3.3 mmHg SBP, no significant effect on DBP
Bondonno et al. (2017) [46]	Apple 48 mg epicatechin, 306 mg total polyphenols	Acute and 4 weeks	Subjects at risk of CVD (*n* = 30)	No significant effect
Grassi et al. (2009) [47]	Black tea 100, 200, 400, 800 mg flavonoids	1 week	Healthy males (*n* = 19)	−2.6 mmHg SBP, −2.2 mmHg DBP
Hodgson et al. (2012) [48]	Black tea 429 mg total polyphenols	6 months	Healthy to mildly hypertensive subjects (*n* = 95)	−2.7 mmHg SBP, −2.3 mmHg DBP
Grassi et al. (2015) [49]	Black tea 258 mg flavonoids	1 week	Hypertensive subjects (*n* = 19)	−3.2 mmHg SBP, −2.6 mmHg DBP
Barona et al. (2012) [52]	Grape 35 mg anthocyanins, 267 mg total polyphenols	1 month	Subjects with metabolic syndrome (*n* = 24)	−6 mmHg SBP, no significant effect on DBP
Rodriguez-Mateos et al. (2013) [53]	Blueberry 766, 1278, 1791 mg polyphenols	Acute	Healthy males (*n* = 10)	No significant effect
Rodrigues-Mateos et al. (2016) [54]	Cranberry 409, 787, 1238, 1534, 1910 mg total polyphenols	Acute	Healthy males (*n* = 10)	No significant effect
Dohadwala et al. (2011) [55]	Cranberry 94 mg anthocyanins, 835 mg total polyphenols	4 weeks	Subjects with CAD (*n* = 44)	No significant effect
Curtis et al. (2009) [56]	Elderberry 500 mg anthocyanins	12 weeks	Postmenopausal women (*n* = 52)	No significant effect
Morand et al. (2011) [57]	Orange juice 292 mg hesperidin	4 weeks	Overweight males (*n* = 24)	−5.5 mmHg DBP, no significant effect on SBP
Rendeiro et al. (2016) [58]	Orange juice 128, 272, 452 mg total flavonoids	Acute	Healthy males (*n* = 28)	No significant effect
Habauzit et al. (2015) [59]	Grapefruit 210 mg naringenin	6 months	Postmenopausal women (*n* = 48)	No significant effect

CAD: coronary artery disease, CVD: cardiovascular disease, DBP: diastolic blood pressure, MCI: mild cognitive impairment, SBP: systolic blood pressure.

**Table 2:** Summary of studies investigating the effect of flavonoids on endothelial function.

Study	Flavonoid Source and Dose	Duration	Sample	Effects
Faridi et al. (2008) [35]	Dark chocolate 821 mg flavanols	Acute	Overweight subjects (*n* = 45)	4.3% increase in FMD
Marsh et al. (2017) [73]	Chocolate 395 mg (dark), 200 mg (milk) total polyphenols	Acute	Postmenopausal women (*n* = 12)	2.4% increase in FMD following dark chocolate, no significant effect of milk chocolate
Schroeter et al. (2006) [72]	Cocoa 917 mg flavanols	Acute	Healthy subjects (*n* = 10)	Increase in FMD
	Pure epicatechin 1 mg/kg, 2 mg/kg body weight	Acute	Healthy subjects (*n* = 3)	Increase in FMD
Dower et al. (2015) [44]	Pure epicatechin 100 mg	4 weeks	Healthy subjects (*n* = 37)	No significant effect
Dower et al. (2016) [42]	Dark chocolate 150 mg epicatechin, 100 mg pure epicatechin with white chocolate	Acute	Healthy males (*n* = 20)	0.96% increase in FMD, no significant effect of pure epicatechin
Engler et al. (2004) [41]	Chocolate 259 mg total flavonoids	2 weeks	Healthy subjects (*n* = 22)	1.3% increase in FMD
Sansone et al. (2015) [32]	Cocoa 900 mg flavanols	1 month	Healthy subjects (*n* = 100)	1.2% increase in FMD
Fisher and Hollenberg (2006) [43]	Cocoa 821 mg flavanols	4–6 days	Healthy younger (aged <50 year, *n* = 15), and older subjects (>50 year, *n* = 19)	3.5% (younger) and 4.5% increase in FMD (older)
Bondonno et al. (2012) [45]	Apples 180 mg epicatechin, 184 mg quercetin	Acute	Healthy subjects (*n* = 30)	1.1% increase in FMD
Bondonno et al. (2017) [46]	Apples 48 mg epicatechin, 306 mg total polyphenols	Acute and 4 weeks	Subjects at risk of CVD (*n* = 30)	0.8% (acute) and 0.5% (chronic) increase in FMD
Saarenhovi et al. (2017) [76]	Apple 100 mg epicatechin	Acute and 4 weeks	Borderline hypertensive subjects (*n* = 60)	No significant effect
Grassi et al. (2009) [47]	Black tea 100, 200, 400, 800 mg flavonoids	1 week	Healthy males (*n* = 19)	2.5% increase in FMD
Schreuder et al. (2014) [69]	Black tea 1800 mg total polyphenols	Acute and 1 week	Healthy subjects (*n* = 20)	1.4% increase in FMD
Jochmann et al. (2008) [77]	Black and green tea 560 mg (black), 1012 mg (green) total catechins	Acute	Postmenopausal women (*n* = 24)	4.4% (black) and 5% (green) increase in FMD
Duffy et al. (2001) [77]	Black tea 964 mg total flavonoids	Acute and 4 weeks	Subjects with CAD (*n* = 50)	4.8% increase in FMD (acute-on-chronic)
Grassi et al. (2016) [79]	Black tea 150 mg polyphenols twice/day	Acute and 8 days	Hypertensive subjects (*n* = 19)	1% (acute) and 1.8% (chronic) increase in FMD
van Mierlo et al. (2010) [81]	Wine and grape seed 800 mg total polyphenols	3 weeks	Healthy males (*n* = 35)	No significant effect
Barona et al. (2012) [52]	Grape 35 mg anthocyanins, 267 mg total polyphenols	1 month	Subjects with metabolic syndrome (*n* = 24)	1.7% increase in FMD
Stein et al. (1999) [82]	Grape	14 days	Subjects with CAD (*n* = 15)	4.2% increase in FMD
Rodriguez-Mateos et al. (2013) [53]	Blueberry 766, 1278, 1791 mg polyphenols	Acute	Healthy males (*n* = 10)	2.4% increase in FMD
Rodriguez-Mateos et al. (2016) [54]	Cranberry 409, 787, 1238, 1534, 1910 mg total polyphenols	Acute	Healthy males (*n* = 10)	2.6% increase in FMD
Dohadwala et al. (2011) [55]	Cranberry 835 mg total polyphenols	4 weeks	Subjects with CAD (*n* = 44)	No significant effect
Rendeiro et al. (2016) [58]	Orange 128, 272, 452 mg total flavonoids	Acute	Healthy males (*n* = 28)	Recovery in % FMD to baseline levels following a high fat meal
Rizza et al. (2011) [83]	Hesperidin 500 mg hesperidin	3 weeks	Subjects with metabolic syndrome (*n* = 24)	2.5% increase in FMD
Habauzit et al. (2015) [59]	Grapefruit 210 mg naringenin	6 months	Postmenopausal women (*n* = 48)	No significant effect

Absolute change in FMD (flow-mediated dilation) response provided where available.

**Table 3:** Summary of studies investigating the effect of flavonoids on cerebral blood flow.

Study	Flavonoid Source and Dose	Duration	Sample	Effects
Francis et al. (2006) [98]	Cocoa 516 mg flavanols	Acute	Healthy adults (aged 24–31 years, *n* = 4)	Increase in CBF across grey matter
Lamport et al. (2015) [102]	Cocoa 494 mg flavanols	Acute	Healthy older adults (aged 50–65 years, *n* = 18)	Increase in regional perfusion (anterior cingulate cortex, central opercular cortex)
Sorond et al. (2008) [104]	Cocoa 450 mg flavanols	1 week	Healthy older adults (aged 59–83 years, *n* = 21)	Increase in cerebral blood flow velocity
Brickman et al. (2014) [105]	Cocoa 900 mg flavanols	3 months	Healthy older adults (aged 50–69 years, *n* = 41)	Increase in cerebral blood volume in the dentate gyrus
Marsh et al. (2017) [73]	Chocolate 395 mg (dark), 200 mg (milk) total polyphenols	Acute	Postmenopausal women (*n* = 12)	Reduction in cerebral blood flow velocity with both dark and milk chocolate
Massee et al. (2015) [40]	Cocoa 250 mg catechin polyphenols	Acute and 4 weeks	Healthy younger adults (aged 18–40 years, *n* = 40)	No significant effect
Dodd et al. (2012) [99]	Blueberry 579 mg flavonoids	Acute	Healthy younger adults (aged 18–25 years, *n* = 19)	Increase in regional perfusion (occipital cortex, frontal lobe, angular gyrus)
Bowtell et al. (2017) [106]	Blueberry 387 mg anthocyanins	12 weeks	Healthy older adults (aged >65 year, *n* = 26)	Increase in regional perfusion (parietal lobe, occipital lobe)
Lamport et al. (2016) [100]	Citrus 70.5 mg flavanones	Acute	Healthy young subjects (aged 18–30 years, *n* = 24)	Increase in regional perfusion (inferior and middle right frontal gyrus)
Wightman et al. (2012) [107]	EGCG 135 mg, 270 mg	Acute	Healthy adults (aged 18–30 years, *n* = 27)	Reduction in CBF to frontal cortex (135 mg), no effect of 270 mg

CBF: cerebral blood flow.
